# Multidisciplinary collaboration successfully treated Budd Chiari syndrome complicated with hepatocellular carcinoma rupture and bleeding: A case report

**DOI:** 10.1097/MD.0000000000046748

**Published:** 2025-12-19

**Authors:** Lian Liao, Yuyang Qiu, Xiaobo Gong

**Affiliations:** a Department of Emergency, The Second People’s Hospital of Guiyang (Jinyang Hospital), Guiyang, Guizhou Province, China; b Department of Hepatobiliary Surgery, The Second People’s Hospital of Guiyang (Jinyang Hospital), Guiyang, Guizhou Province, China.

**Keywords:** Budd-Chiari syndrome, hepatic venous outflow tract obstruction, hepatocellular carcinoma, multidisciplinary diagnosis and treatment, surgery

## Abstract

**Rationale:**

Budd-Chiari syndrome (BCS) complicated by ruptured hepatocellular carcinoma (HCC) is relatively uncommon. This case underscores the critical role of multimodal imaging and interdisciplinary collaboration.

**Patient concerns:**

A 46-year-old female presented with abdominal pain for 10 + days, aggravated for 5 hours. the emergency abdominal enhanced computed tomography: malignant space occupying in the left inner lobe of liver (HCC is possible) and hematocele in abdominal cavity. Magnetic resonance imaging enhancement of the upper abdomen was performed: malignant mass of the left lobe of the liver with hemorrhage and necrosis, compensatory enlargement of the right lobe of the liver, unclear display of hepatic veins, and stenosis of the hepatic segment of the inferior vena cava, which was consistent with Budd-Chiari syndrome.

**Diagnoses:**

Budd-Chiari syndrome with hepatocellular carcinoma rupture and bleeding.

**Interventions:**

Interventional therapy, surgical treatment.

**Outcomes:**

Hepatic venous outflow obstruction was successfully relieved by interventional therapy combined with surgery and tumor resection was performed. After 39 days of intensive care, the patient improved and was discharged smoothly.

**Lessons:**

For patients with Budd-Chiari syndrome and hepatocellular carcinoma, the Multidisciplinary diagnosis and treatment (MDT) model should be implemented throughout the entire treatment process. Through the MDT model, personalized treatment plans can be developed, combining various methods to relieve liver vein outflow obstruction and remove tumors, while also establishing appropriate treatment measures and processes, thereby improving treatment outcomes and reducing the risk of complications and disease progression. However, within the MDT model, different physicians must propose treatment plans based on their professional knowledge and the specific circumstances of each patient. In the process of personalized treatment, issues such as how to balance pros and cons and select the optimal solution also present challenges to clinical practitioners.

## 1. Introduction

Budd-Chiari syndrome (B-CS) is a rare hepatic vascular disease characterized by obstruction of blood flow in the hepatic segment of the hepatic vein and/or the inferior vena cava, and its pathophysiological changes are mainly manifested as hepatic stasis, portal hypertension, and secondary hepatic impairment due to obstruction of hepatic venous return.^[1]^ With the rapid development of diagnostic imaging technology and the deepening of clinicians’ understanding of this disease, the clinical detection rate of B-CS has shown a significant increase in recent years. However, the clinical cases of B-CS combined with ruptured bleeding of hepatocellular carcinoma (HCC) are still rare, and its rapid progression and poor clinical prognosis have made it one of the most challenging critical illnesses in the field of hepatobiliary surgery. As the most serious complication of HCC, hepatic rupture bleeding has a very high lethality rate. Notably, patients with B-CS have a significantly higher risk of HCC rupture hemorrhage and greater difficulty in hemorrhage control due to liver parenchymal injury caused by prolonged hepatic stasis, coagulation dysfunction associated with portal hypertension, and underlying vascular abnormalities, which further exacerbates the complexity of clinical treatment and the risk of patient death. In this study, we report a case of B-CS combined with ruptured bleeding of HCC that was successfully treated by multidisciplinary diagnosis and treatment (MDT), which is intended to provide a reference for the clinical diagnosis and treatment of such critical cases.

## 2. Case presentation

A 46-year-old female presented with abdominal pain for 10+ days, aggravated for 5 hours. There was no specific past. A physical examination was performed upon admission on September 20, 2023. The body temperature was 36.8°C, heart rate was 99 beats/min, breathing rate was 19 beats/min, and blood pressure was 91/53 mm Hg (1 mm Hg = 0.133 kPa). Cardiopulmonary examination showed no significant abnormalities. The abdomen was flat and soft, with scattered tenderness throughout the abdomen and no rebound pain or muscle tension. There was tenderness to percussion in the hepatic region, and abdominal percussion was turbid, with positive mobile turbidity. Bowel sounds were present, about 2 beats/min. Mild depressed edema of both lower extremities.

**Figure 1.**
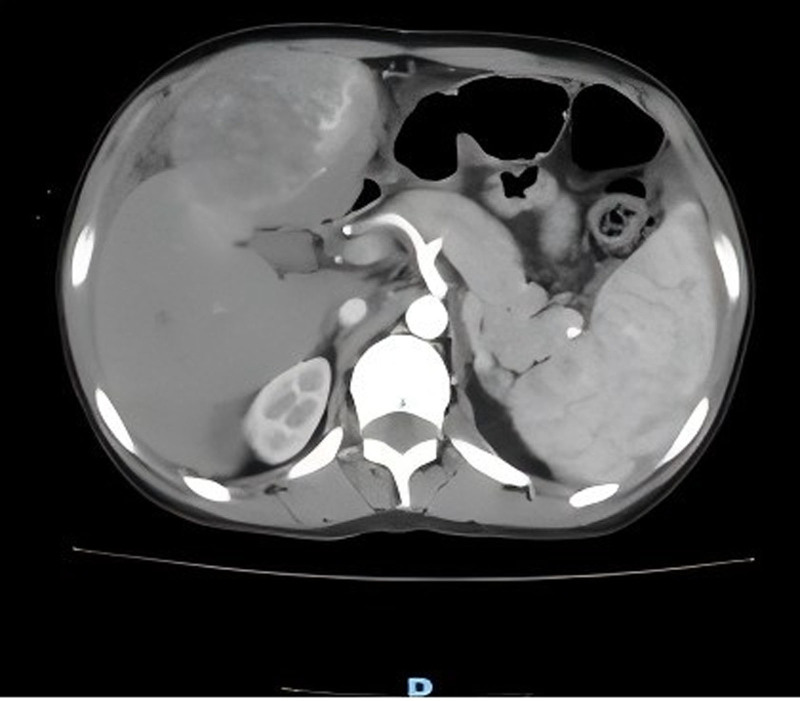
Abdominal enhanced CT: malignant mass in left medial lobe of liver (HCC possible), hemoperitoneum. CT = computed tomography, HCC = hepatocellular carcinoma.

**Figure 2.**
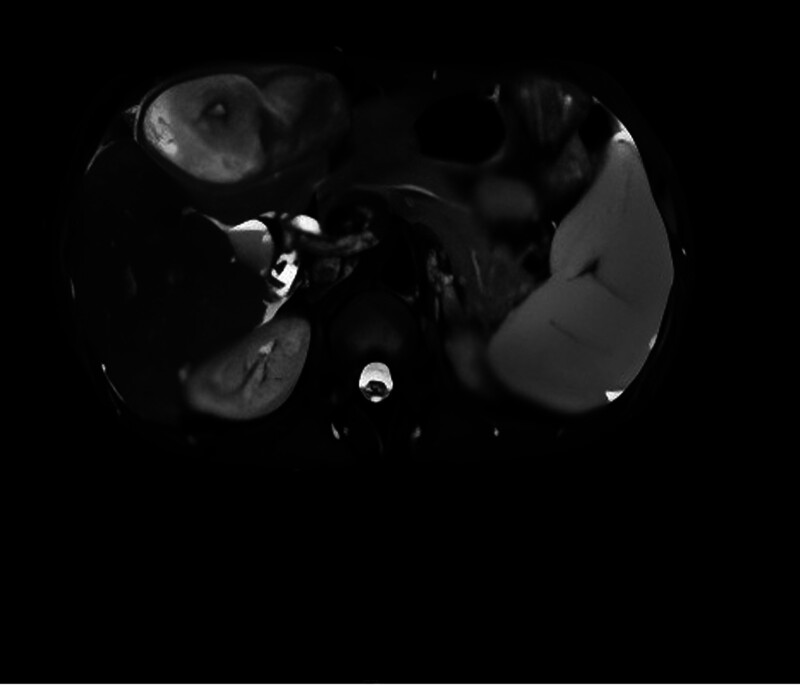
Epigastric MRI plain + enhanced + MRCP + DWI: malignant mass with hemorrhagic necrosis in left lobe of liver, compensatory enlargement of right lobe of liver, unclear display of hepatic veins, hepatic segment stenosis of inferior vena cava, consistent with Budd-Chiari syndrome. DWI = diffusion weighted imaging, MRCP = magnetic resonance cholangiopancreatography, MRI = magnetic resonance imaging.

After admission, the patient’s circulation was unstable (blood pressure fluctuated between 85–100/44–60 mm Hg) and hemoglobin dropped to 80 g/L. Diagnostic abdominal puncture draws out dark red blood without coagulation. Improve the emergency abdominal enhanced computed tomography (CT) (Fig. 1): malignant space occupying in the left inner lobe of liver (HCC is possible) and hematocele in abdominal cavity. Emergency left hepatic artery embolization was performed on the same day, and the blood pressure was stable (105–115/60–70 mm Hg) and hemoglobin was maintained at 92 to 94 g/L. On the third day after operation, the abdominal drainage decreased from 1340 to 350 mL/d and changed from bloody to pale yellow. On October 8th, magnetic resonance imaging enhancement of the upper abdomen was performed (Fig. 2): malignant mass of the left lobe of the liver with hemorrhage and necrosis, compensatory enlargement of the right lobe of the liver, unclear display of hepatic veins, and stenosis of the hepatic segment of the inferior vena cava, which was consistent with Budd-Chiari syndrome. Laboratory examination: alpha fetal protein (AFP) > 1210.0 ng/mL, alanine aminotransferase (ALT) 68 U/L, aspartate aminotransferase (AST) 75 U/L, total bilirubin 25.3 μmol/L, albumin 32 g/L, prothrombin time 15.2 seconds. Hepatitis markers are negative. Chest CT: interstitial inflammation, azygos vein dilatation, and anemia in the lower lobe of both lungs.

MDT discussion on October 12th: Budd-Chiari syndrome complicated with rupture and bleeding of HCC was diagnosed, and the operation was decided by stages. On October 16, balloon angioplasty of inferior vena cava was performed first. On October 19th, partial hepatectomy + cholecystectomy + intestinal adhesion release was performed: during the operation, a hard mass of 10 × 15 cm was found in the left lobe of the liver, with mild adhesion and bleeding of about 100 mL.

**Figure 3.**
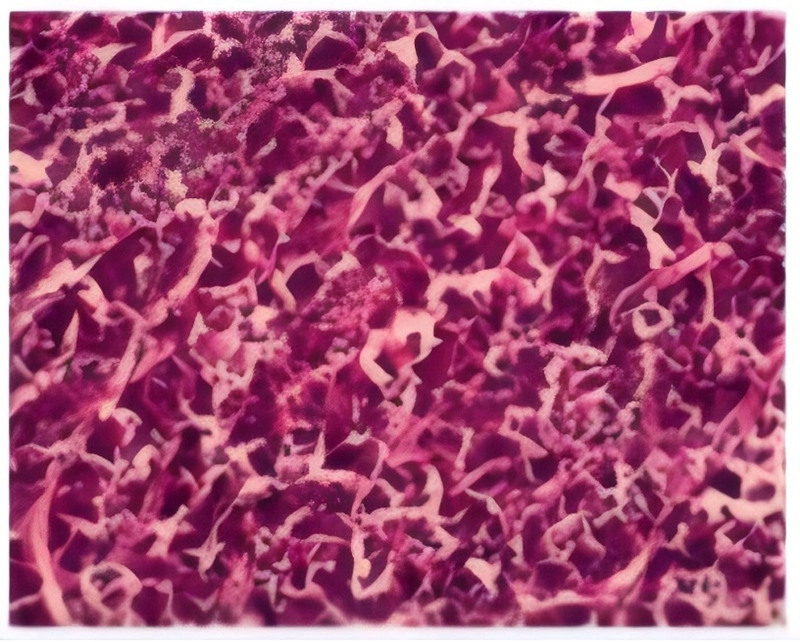
Pathological biopsy: “S4b liver tumor” moderately differentiated hepatocellular carcinoma, nodular type, with tumor involvement of the hepatic peritoneum, suspected intravascular embolus, no neurological invasion, and no carcinoma involvement of the hepatic margins. “The gallbladder is not involved.”

**Figure 4.**
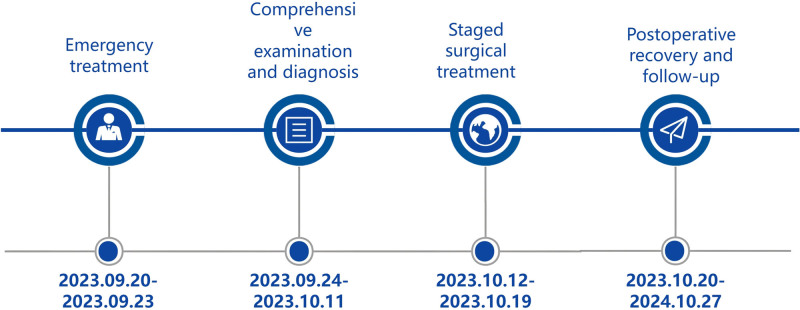
The time of medical history.

On the first day after operation: ALT65U/L, AST187U/L, total bilirubin 32.1 μmol/L. On the third day after operation, eating resumed, and liver function improved (ALT49U/L, AST50U/L, total bilirubin 20.7 μmol/L). The abdominal drainage tube was removed on the fifth day after operation. On the 10th day after operation, the incision healed well, and all the indexes were discharged normally. Pathological examination (Fig. 3): Hepatocellular carcinoma (immunohistochemistry: CK19+, AFP+, Hepatocyte+, Glypican-3+). Ranvartinib targeted therapy was started one month after operation. One-year follow-up: no recurrence or metastasis, AFP decreased to 28.4 ng/mL, and liver function was normal (Fig. 4). The time of medical history.

## 3. Conclusions and lessons

B-CS is a clinical syndrome characterized by varying degrees of hepatic venous outflow tract obstruction from the small hepatic veins to the inferior vena cava in the absence of stenosing pericarditis or right heart failure, resulting in portal and/or inferior vena cava hypertension.^[2,3]^ Currently, B-CS is classified into primary hepatic venous obstruction, inferior vena cava obstruction, and mixed types based on the location of the obstruction. The incidence of B-CS is distinctly geographic. In Asian countries such as China and Japan, the inferior vena cava obstructive type is predominantly manifested, while the primary hepatic venous obstruction type is predominant in Western countries.^[1]^ According to the imaging data of this patient, the inferior vena cava obstruction type can be considered. Patients with B-CS have a slow and insidious onset, with a history of several years or even decades, with a long asymptomatic period, and in a few cases, an acute onset. They most often present with symptoms such as lower extremity edema and massive abdominal effusion.^[3–5]^ As the disease progresses, patients will gradually develop HCC-related clinical manifestations, such as fatigue, emaciation, pain in the liver region, jaundice, pleural and abdominal effusions, or palpable abdominal masses, and liver failure or even shock in severe cases. If HCC rupture occurs, severe abdominal pain, hemorrhagic shock and other acute manifestations will occur. In this case, the patient was admitted to the hospital with the main symptoms of abdominal pain, percussion pain in the liver area, and hemorrhagic shock. Perfect examination considered HCC rupture and bleeding, and the condition gradually stabilized after emergency interventional hemostasis treatment. The diagnosis of B-CS mainly relies on imaging examinations, including ultrasound, CT venography, MR, DSA, etc.^[1]^

HCC is one of the most common cancers in the world, with extremely high mortality rate and insidious onset of disease, and for patients with early-stage HCC, the curative treatment is tumor resection. However, early diagnosis and treatment of HCC is difficult; the disease progresses rapidly and has a poor prognosis.^[6]^ Early definitive diagnosis is crucial, as most patients reach advanced stages when detected, but treatment options for advanced HCC are limited and require multidisciplinary collaboration to determine, as the incidence of HCC continues to increase and mortality remains high. However, the differential diagnosis between HCC and benign liver lesions is difficult, and biochemical markers and imaging tests are often required to assist in the diagnosis. Elevated serum AFP is an important basis for the diagnosis of HCC in patients with B-CS. Previous findings have shown that elevated AFP has a very high specificity in identifying benign and malignant lesions in the liver of B-CS patients.^[7]^ It has also been pointed out that some patients with benign liver disease have overexpression of AFP, which makes the specificity of AFP for detecting HCC affected. In addition, the diagnostic sensitivity of AFP is affected by the size of the tumor.^[6]^ Therefore, the diagnostic value of AFP is still controversial and needs to be verified by multicenter and large-sample clinical studies. HCC complicated by B-CS mostly manifests as a single, large, irregular nodule located in the periphery of the liver but partially lacks HCC-specific imaging features. Due to the obstruction of hepatic outflow tracts in B-CS patients, some patients with B-CS-associated HCC do not have the typical “fast in, fast out” signs, which can be easily confused with hepatic atypical hyperplasia nodules and misdiagnosed. Therefore, the diagnostic value of diagnostic imaging is limited in patients with B-CS, and the diagnosis of B-CS is still challenging, and ultimately, it may need to be performed for diagnostic purposes by aspiration biopsy. Puncture biopsy is the gold standard for the diagnosis of HCC complicated by B-CS. HCC complicated by B-CS is often well differentiated, with a predominance of highly differentiated tumors that are less invasive. Tumors invade peripheral blood vessels and bile ducts less frequently, and the surrounding tissues usually show severe fibrosis or cirrhosis. Postoperative pathologic biopsy of our patient: no nerve invasion, no hepatic incision margin, or gallbladder involvement. Reticulofibrillar staining and detection of immunophenotypic markers of malignancy are helpful in the diagnosis of HCC.^[1]^ Some researchers found that Glypican-3 mRNA and protein levels were increased in 74.8% of HCC patients, but not expressed or expressed at low levels in benign liver disease patients and normal subjects.^[8,9]^ This suggests that the abnormal expression of Glypican-3 is closely related to the development and malignancy of HCC. Glypican-3 is positive in this patient, which can assist in the diagnosis, treatment, and prognosis of HCC.

The treatment of B-CS complicated with HCC is in addition to maintaining organ function and internal environment stability. It is necessary to consider the treatment of both B-CS and HCC at the same time, not only to solve the sludge state of the liver, but also to resect the tumor or take other measures to slow down the progression of the tumor. Therefore, a patient-centered MDT diagnosis and treatment model should be constructed to give full play to the advantages of various disciplines, avoid the limitations of a single discipline, choose individualized treatment modalities according to the patient’s physical condition and the different stages of the tumor, solve the patient’s difficulties in diagnosis and treatment, and maximize the benefits to the patient as much as possible in order to improve the overall treatment effect. For B-CS combined with HCC, relief of hepatic venous outflow tract obstruction by interventional or surgical means is particularly important in the treatment of B-CS complicated with HCC, which can restore hepatic blood flow, alleviate hepatic stagnation and hypoxic microenvironment, which can delay tumor progression and reduce complications. Surgical resection, transcatheter arterial chemoembolization, local tumor ablation, or liver transplantation are chosen according to the patient’s condition. Among them, surgical resection of the tumor is an important means to prolong the survival of patients with B-CS complicated with HCC. For patients with Child-Pugh classification of A or B, hepatic resection remains an effective and optimal choice for the treatment of B-CS complicated with HCC.^[10]^ Treatment choices may vary from case to case, and treatment strategies need to be adapted to the individual patient. In our case, we first intervened to relieve the hepatic venous outflow tract obstruction and then adopted surgical resection as a treatment tool according to the HCC, with regular postoperative follow-up for 1 year, with improvement in liver function and jaundice, and no recurrence or metastasis.

MDT mode runs through the whole diagnosis and treatment process of this patient with B-CS complicated with HCC. Through dynamic evaluation of efficacy and timely adjustment of treatment regimen, it realizes individualized and precise treatment. MDT collaboration helps to promote multidisciplinary clinical practice of this complex disease. It not only provides important ideas for the diagnosis and treatment of B-CS complicated with HCC but also lays a solid foundation for formulating standardized diagnosis and treatment procedures, improving treatment benefits, and reducing complications and disease progression risks. It is an important guarantee for ensuring medical quality and safety.

## Author contributions

**Conceptualization:** Yuyang Qiu.

**Data curation:** Xiaobo Gong, Yuyang Qiu.

**Formal analysis:** Yuyang Qiu.

**Funding acquisition:** Xiaobo Gong.

**Supervision:** Xiaobo Gong.

**Writing – original draft:** Lian Liao.

**Writing – review & editing:** Lian Liao.
